# CD14+ Monocytes Will Become a New Target for the Treatment of Osteoporosis: Based on Mendel Randomization, Clinical Analysis and Cell Experiment Verification

**DOI:** 10.1111/jcmm.71024

**Published:** 2026-01-23

**Authors:** Haoran Wang, Xiao Ma, Ping Zhou, Jie Zhang, Boyao Wang, Jun Liu

**Affiliations:** ^1^ Department of Orthopaedics, Tongji Hospital of Tongji University, School of Medicine Tongji University Shanghai China; ^2^ Department of Oncology The Second Affiliated Hospital of Nanjing Medical University Nanjing China; ^3^ Lab Center The Second Affiliated Hospital of Nanjing Medical University Nanjing China; ^4^ Department of Oncology The First People's Hospital of Lianyungang Lianyungang China; ^5^ Department of Orthopaedics The Second Affiliated Hospital of Nanjing Medical University Nanjing China

**Keywords:** CD14, Mendel randomization, monocyte, osteoblast, osteoclast, osteoporosis

## Abstract

To explore the causal relationship between monocytes and osteoporosis by Mendel randomization, and to verify it through subsequent experiments. Data regarding osteoporosis and immune cell phenotypes were sourced from the GWAS‐Catalogue database. We utilised several Mendelian randomization methods, including the inverse variance weighted method, MR‐Egger, weighted median method, and simple median method, complemented by Cochran's Q, MR‐Egger regression and Leave‐One‐Out analysis. Clinical samples were classified into healthy and osteoporosis groups, and blood samples from both cohorts were analysed using flow cytometry. In vitro cell experiments were performed to investigate the effect of si‐CD14 on the differentiation of monocytes into osteoclasts, employing western blotting, qPCR and TRAP staining techniques. In addition, we assessed the impact of CD14+ monocytes on the proliferation and mineralisation of osteoblasts through western blotting, qPCR and Alizarin Red staining, and further investigated the underlying mechanisms. Cochran's Q results indicated that the Mendelian randomization findings exhibited heterogeneity; therefore, the conclusions of this study were derived from the inverse variance weighting method. The weighted results of this method demonstrated a positive causal relationship between CD14+ monocyte count and osteoporosis (β = 0.096599, 95% CI: 1.06246, 1.141806, *p* = 1.46E−07). Additionally, the CD14+/CD16− monocyte count was found to have a positive causal relationship with osteoporosis (β = 0.097927, 95% CI: 1.065098, 1.142008, *p* = 3.67E−08). Mouse monocytes are activated through the NF‐kB pathway under RANKL stimulation, leading to their differentiation into osteoclasts; however, si‐CD14 transfection can inhibit this differentiation. Similarly, glucocorticoid stimulation can inhibit the proliferation and mineralisation of osteoblasts, while co‐culturing with CD14+ monocytes exacerbates the glucocorticoid‐induced biological activity, which is regulated by the TGF‐β/SMAD3 pathway. Increased levels of CD14+ monocytes or CD14+/CD16− monocytes are recognised as risk factors for osteoporosis. CD14 plays a crucial role in this process. Inhibition of CD14 expression in monocytes can prevent their differentiation into osteoclasts by suppressing the NF‐kB pathway. Additionally, the co‐culture of CD14+ monocytes with osteoblasts has been shown to inhibit the TGF‐β/SMAD3 pathway, thereby suppressing the proliferation and mineralisation of osteoblasts.

## 1 Introduction

Osteoporosis (OP) is a disease characterised by osteopenia and increased bone fragility resulting from the deterioration of bone microstructure. The most severe complication associated with OP is brittle fracture, which significantly impacts the quality of life of affected individuals [1]. With the increasingly evident trend of global population ageing, it is estimated that over 200 million individuals worldwide will be affected by OP [2]. It is of great significance to explore the biological changes at the level of OP gene for the treatment and prevention of OP.

Osteoblasts and osteoclasts are crucial in the onset and progression of osteoporosis. Recent studies have demonstrated a significant relationship between peripheral blood immune cells and OP. These immune cells interact with osteoblasts and osteoclasts through direct cell‐to‐cell contact, as well as autocrine and paracrine mechanisms, thereby influencing bone metabolism [3, 4]. Moreover, osteoclasts are differentiated terminal cells, which have no ability to proliferate and divide, and are differentiated from monocytes in vivo [5]. Monocytes can be divided into three types: CD14+/CD16−, CD14+/CD16+ and CD14−/CD16+ [6]. Historically, there has been a lack of comprehensive studies examining the transformation of various types of monocytes into osteoclasts, as well as limited research on their influence on the proliferation and mineralisation of osteoblasts. OP is a chronic disease that typically goes undiagnosed until a patient experiences a fracture; therefore, early intervention is crucial for the prevention and treatment of osteoporosis [7, 8]. Currently, the research on the relationship between monocytes and OP relies predominantly on observational studies. Such studies are susceptible to various confounding factors and reverse causality, which may compromise the accuracy of the conclusions drawn.

Mendelian randomization is an innovative research method that leverages the principles of Mendelian genetics. This approach employs multiple or single nucleotide polymorphisms, which are not influenced by confounding factors, as instrumental variables to assess the impact of exposure factors on disease risk. The primary advantage of this method lies in its ability to mitigate the effects of confounding variables, thereby offering more robust evidence to support causal relationships in disease aetiology [9]. Exploring the causal relationship between different types of monocytes and OP may provide crucial insights for early diagnosis and intervention strategies for the disease. Accordingly, this study employs bidirectional Mendelian randomization analysis, supplemented by clinical samples and cytological experiments, to investigate the genetic basis of the relationship between CD14+ monocytes or CD14+CD16− monocytes and OP.

## 2 Materials and Methods

### 2.1 Data Source

The peripheral blood mononuclear cell data, identified as GCST90001991, is derived from the GWAS meta‐analysis conducted by Orrù et al. in 2020, which included a cohort of 3629 European individuals. The dataset GCST90001476 comprises 3595 European participants, while GCST90001988 also encompasses a total of 3629 European individuals. The OP‐related data was sourced from the Finngen database, comprising 7300 patients diagnosed with OP and 358,014 controls, resulting in a total of 9,587,836 single nucleotide polymorphisms (SNPs). Since this research utilises publicly available data, no additional ethical approval or consent is necessary.

The data for OP patients in this study was sourced from the GSE35958 and GSE35959 datasets in the NCBI GEO database, utilising the Affymetrix Human Genome U133 Plus 2.0 Array platform (GPL570).

All clinical samples and case data were obtained from the Department of Orthopaedics at the Second Affiliated Hospital of Nanjing Medical University. Diagnostic criteria of OP: normal: *T*‐value ≥ −1.0; Low bone mass: −2.5 < *t*‐value < −1.0; Osteoporosis: *T*‐value ≤ −2.5; Severe osteoporosis: *T*‐value ≤ −2.5 [10]. This study received approval from the Ethics Committee of the Second Affiliated Hospital of Nanjing Medical University, with the ethical document number 2025‐KY‐002‐01.

### 2.2 Tool Variable Selection

Mendelian randomization is founded on three key hypotheses: (1) genetic variation is significantly associated with the exposure of interest; (2) genetic variation is independent of confounding factors; and (3) genetic variation influences the outcome solely through its effect on the exposure. Based on these hypotheses, our criteria for selecting instrumental variables are as follows: we identify single nucleotide polymorphisms (SNPs) that exhibit a strong correlation with the exposure factors through correlation analysis, with a screening threshold of *p* < 5e−08. We also exclude SNPs that are in linkage disequilibrium (LD) with an *r* ^2^ < 0.001 and a distance greater than 10,000 kb. Furthermore, weak instrumental variables are discarded based on a threshold of *F* statistics > 10 to mitigate the influence of weak instruments. The formula for calculating *F* statistics is *F* = *R* ^2^ × (*N* − 2)/(1 − *R* ^2^). Finally, we address confounding factors by identifying and removing them, along with their associated SNPs, using the PhenoScanner database (http://www.phenoscanner.medschl.cam.ac.uk/) [11, 12].

### 2.3 Mendel Randomization Analysis

Utilising the inverse variance weighting method as the primary analytical approach offers significant advantages due to its simplicity and efficiency. This method is particularly effective when each gene variation serves as a valid instrumental variable, meaning it is strongly associated with exposure factors and influences the results solely through these exposure factors [13, 14]. To mitigate the impact of horizontal pleiotropy on the accuracy of the inverse variance weighting method, we employed MR‐Egger and the weighted median as supplementary methods for causal inference, which can account for potential polymorphism [15]. When OP or monocyte count is the outcome, the effect size is represented as a continuous variable, with β and 95% confidence intervals (CI) employed to assess the causal relationship between peripheral blood mononuclear cells and OP. Cochran's Q statistic was utilised to evaluate the heterogeneity among single nucleotide polymorphisms associated with OP in peripheral blood mononuclear cells. The decision to apply a random effects model or a fixed effects model was based on the presence of heterogeneity, determined by a *p*‐value threshold of 0.05 (*p* < 0.05 indicates heterogeneity). Additionally, potential horizontal pleiotropy was assessed using the MR‐Egger regression intercept, with a *p*‐value of < 0.05 suggesting the presence of horizontal pleiotropy.

Leave‐one‐out method is used to test the sensitivity of the results [16]. This method evaluates whether individual single nucleotide polymorphisms (SNPs) influence the estimated values derived from the inverse variance weighting method by sequentially eliminating each SNP. The aforementioned statistical analyses are conducted using the ‘Two‐Sample‐MR’ and ‘MR‐PRESSO’ packages within RStudio. When either the outcome is operationalised as OP or peripheral blood mononuclear cells, the effect sizes are represented as continuous variables, denoted by β and 95% confidence intervals (CI). The analyses are performed using RStudio software and the ‘TwoSampleMR’ package.

### 2.4 Cell Experiment

Mouse monocyte macrophage cell line RAW264.7 (STCC20020P), obtained from the Zishan Biotechnology Co. Ltd. (Wuhan, China), was cultured in a complete medium consisting of 10% foetal bovine serum (Lonsera) and 1% penicillin/streptomycin (HyClone, GE). The culture was maintained in an incubator at 37°C with 5% CO_2_. Once the fusion rate of the mononuclear macrophages reached 85%, they were either subcultured or utilised for subsequent experiments, and were plated in 24‐well plates at a density of 1 × 10^6^ cells per well [17]. The following day, mononuclear macrophages were treated with RANKL (NF‐κB receptor activator) at a working concentration of 100 μg/mL (GC26626, GLPBIO, China). If necessary, mononuclear macrophages were transfected with si‐CD14 (MTZ‐202411, GENE Biotechnology, China). Cells were harvested 24 h later for further analysis [18].

Mouse osteoblasts (MC3T3‐E1) (STCC20026) were obtained from the Zishan Biotechnology Co. Ltd. (Wuhan, China), and cultured under the same conditions as mentioned above. Osteoblasts were treated with glucocorticoids (GC) at a working concentration of 0.2 mg/mL (HY‐14648, MCE, China). When necessary, CD14+ monocytes were co‐cultured with osteoblasts in Transwell experiments. Cells were harvested after 24 h for further analysis [19, 20].

### 2.5 qPCR

RT‐PCR was employed to assess the expression changes of TRAP mRNA and NFATc1 mRNA during the differentiation of monocytes into osteoclasts. Total RNA from cells in each group was extracted using the Super Fast Pure Cell RNA Isolation Kit (RC 102, Vazyme, China), and cDNA of mRNA was synthesised using HIScript ‖ Reverse Transcriptase (R223, Vazyme, China). The primer design and reaction system were as follows: *Acp5* upstream primer: 5′‐TGGATTCATGGGTGGTGCTG‐3′, *Acp5* downstream primer: 5′‐AGCCACAAATCTCAGGGTGG‐3′; *NF‐ATc1* upstream primer: 5′‐CTGCAACAAGCGCAAGTACA‐3′, *NF‐ATc1* downstream primer: 5′‐AGGTCCAGAGTGCTATCGGT‐3′. *Actb* served as the internal reference, with upstream primer 5′‐TTGCCGACAGGATGCAGA‐3′ and downstream primer 5′‐GCGCGATCCACAGGAGTACT‐3′. Concurrently, the expression levels of OPN mRNA and Collagen‐I mRNA in osteoblasts were evaluated. The primer design and reaction system were as follows: *Spp1* upstream primer: 5′‐CACATGAAGAGCGGTGAGTCT‐3′, *Spp1* downstream primer: 5′‐CCCTTTCCGTTGTTGTCCTG‐3′; *COLIA1* upstream primer: 5′‐AGCACGTCTGGTTTGGAGAG‐3′, *COLIA1* downstream primer: 5′‐GACATTAGGCGCAGGAAGGT‐3′. Primers were synthesised by Nanjing Kingsrui Biotechnology Co. Ltd. The following 10 μL reaction system was prepared in an octuple tube: 5 μL of 2× ChamQ Blue Universal SYBR qPCR Master Mix (Q312, Vazyme, China), 0.2 μL of Primer 1 (10 μM), 0.2 μL of Primer 2 (10 μM), 2 μL of cDNA and 2.6 μL of ddH_2_O. The reaction procedures were set on the fluorescence quantitative PCR instrument as follows: 95°C for 30 s, followed by 40 cycles of 95°C for 10 s and 60°C for 30 s. Each sample was run in triplicate, and the amplification curve was generated automatically by the computer, recording the cycle threshold (Ct). The relative expression of the target gene IL‐33 mRNA was calculated using the 2^−ΔΔCt^ [21].

### 2.6 Western Blot Analysis

Proteins from osteoblasts and mononuclear macrophages were extracted using an immunoprecipitation analysis buffer and subsequently analysed by Western blotting. Equal volumes of the samples were loaded onto a 10% sodium dodecyl sulphate/polyacrylamide gel. The proteins were then separated via electrophoresis and transferred to a polyvinylidene fluoride (PVDF) membrane. To prevent nonspecific binding of antibodies to antigens, the membrane was immersed in 5% bovine serum albumin at 27°C for 1 h. Following this, the membrane was washed three times with PBS containing Tween. Subsequently, the membrane was incubated with Anti‐CD14 antibody (CB11254, Servicebio, China), Anti‐TRAP (GB113426, Servicebio, China), anti‐NFATc1 (GB 11027, Servicebio, China), Anti‐OPN (GB112328, Servicebio, China) and Anti‐Collagen‐I (GB 115707, Servicebio, China), Anti‐Phospho‐NF‐kB p65 (S536) Rabbit pAb (GB113882, Servicebio, China), Anti‐NF‐κB p65 Rabbit pAb (GB11997, Servicebio, China), Anti‐P‐IκBα (9246S, Cell Signalling Technology, USA), Anti‐IκBα (4814P, Cell Signalling Technology, USA), P‐Smad3 80427‐2‐RR (Proteintech, China) and Smad3 (66516–1‐lg, Proteintech, China). Additionally, Anti‐β‐actin antibody (4970, Cell Signalling, USA) was employed to standardise the expression of all proteins [22]. The next day, the primary antibody was first collected, and the PVDF membrane was then retrieved and washed 3–5 times with TBST. The membrane was incubated with the HRP‐conjugated secondary antibody corresponding to the species of the primary antibody at room temperature on a shaking incubator for 1–2 h. After recovering the secondary antibody and removing the PVDF membrane, it was washed 3–5 times with TBST. ECL chemiluminescent reagent was added for exposure, and ImageJ software was used for quantitative analysis of grey values.

### 2.7 TRAP Staining

Absorb the cell culture solution and add 4% paraformaldehyde to fix the cells for 15 to 30 min. Subsequently, wash the cells three times with distilled water. Next, cover the cells with a 0.2% Triton X‐100 solution to permeabilize the membrane for 20 to 30 min, followed by three washes with distilled water. Afterward, add TRAP solution (G1050, Servicebio, China) to the cell culture plate, ensuring the cells are fully covered. Incubate the cells at 37°C in the dark for 15–20 min and wash them three times with distilled water. Finally, absorb the incubation solution, wash with water and observe the cells under a microscope after staining with haematoxylin dye solution [23].

### 2.8 Alizarin Red Staining

Remove the medium from the culture plate and wash it with phosphate‐buffered saline (PBS) three times. Add 4% paraformaldehyde and fix the cells for 15–30 min, followed by three washes with distilled water. Slowly add Alizarin Red S dye solution (G1038, Servicebio, China) until the cells are completely covered, and stain at room temperature for 30 min. Rinse gently with distilled water until no dye remains in the culture plate, then observe and photograph the cells under a microscope [24].

### 2.9 Flow Detection

To extract 1 mL of venous blood, use an EDTA‐K2 anticoagulant vacuum blood collection tube, and ensure that the specimen tube is properly labelled. Subsequently, add 2 μL of CD14‐APC (E‐AB‐F1209E, Elabscience, China), CD16‐PE (E‐AB‐F1236D, Elabscience, China) and FITC Anti‐Human HLA‐DR (E‐AB‐F1111C, Elabscience, China) into the tube. Following this, introduce 50 μL of whole blood into the tube and mix thoroughly. Incubate the mixture in the dark for 15 min at room temperature. After incubation, add 1 mL of FACSLysing to lyse the blood cells, and continue to incubate in the dark for an additional 15 min at room temperature. The cells should then be washed twice with PBS, centrifuged at 300 g for 5 min, and subsequently resuspended after discarding the supernatant, prior to detection using a computer [25].

### 2.10 Statistical Analysis

SPSS 22.0 statistical software was used for statistical analysis. According to the actual situation, the measurement data are expressed by the mean standard error (difference) [*x* ± SEM(SD)] *T* test or one‐way ANOVA is used for comparison between groups, and Tukey method is used for comparison between pairwise, and the difference is statistically significant (*p* < 0.05). The work has been reported in line with the ARRIVE guidelines 2.0.

## 3 Result

**FIGURE 1:**
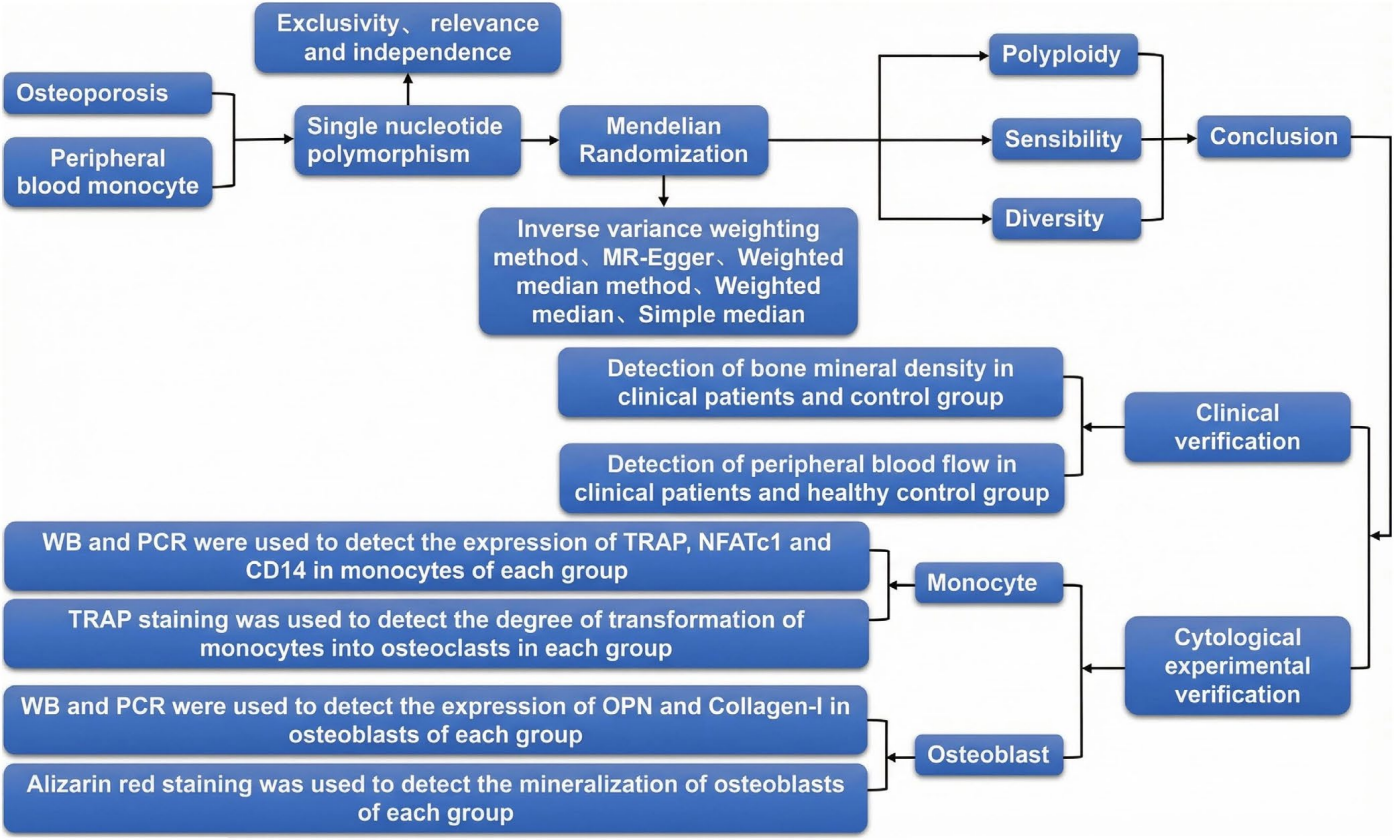
Research process. This study employs a two‐sample bidirectional Mendelian randomization approach to investigate the causal relationship between peripheral blood CD14+ monocytes and CD14+/CD16− monocytes with OP. Clinical data corroborate the pivotal role of CD14+/CD16− monocytes in the onset and progression of OP, leading to the inference of the significance of the CD14 gene in the regulation of OP. Further cellular experiments confirm that CD14 can modulate the process of monocyte differentiation into osteoclasts, and co‐culturing CD14+ monocytes with osteoblasts can inhibit the mineralisation process of osteoblasts.

The two‐sample bidirectional Mendelian randomization method was employed to investigate the causal relationship between CD14+ monocytes and CD14+/CD16− monocytes in relation to OP. Furthermore, the analytical results were corroborated through supplementary clinical and cytological experiments (Figure 1).

### 3.1 Tool Variables of CD14+ Monocytes and CD14+/CD16− Monocytes on OP

After removing the linkage disequilibrium single nucleotide polymorphisms, the MR‐PRESSO analysis indicated that there were no outliers, with all single nucleotide polymorphisms satisfying the *F*‐statistic requirement of > 10. Consequently, all single nucleotide polymorphisms adhered to both the independence and exclusivity hypotheses.

### 3.2 Mendel Randomised Analysis Results

**FIGURE 2:**
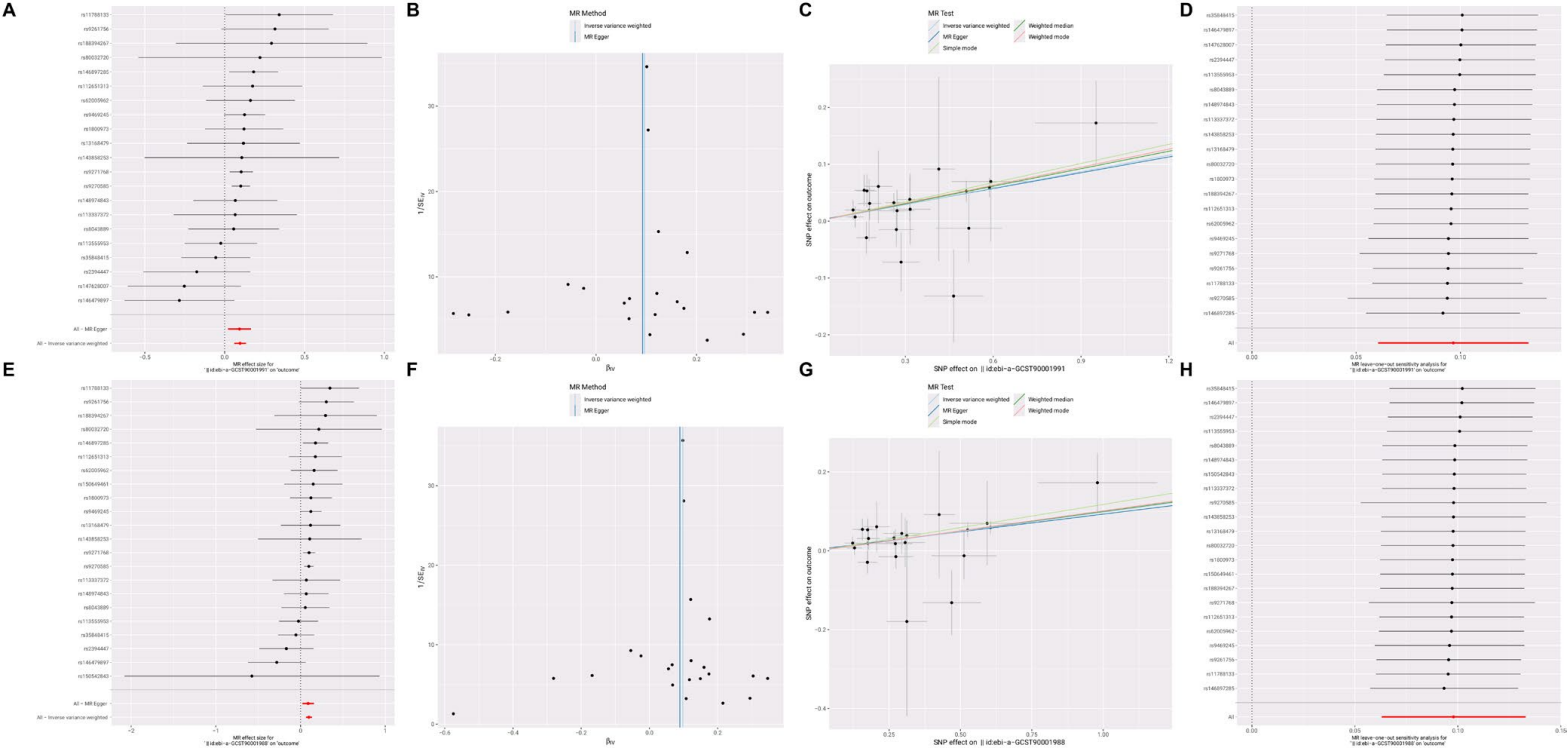
Mendel randomised analysis results. (A) Black dots represent a single SNP in estimating the effect of CD14+ monocyte on the risk of osteoporosis; the red dot represents the overall effect of CD14+ monocyte on osteoporosis estimated by MR‐Egger method and IVW method. (B) The black dot represents a single nucleotide polymorphism, and the SNP in the funnel diagram is basically symmetrical, so the research results are less affected by bias. (C) The abscissa indicates the influence of a single SNP on osteoporosis, and the ordinate indicates the influence of a single SNP on CD14+ monocyte. The colour line is the fitting line of the estimated causal influence of CD14+ monocyte on osteoporosis. (D) Black dots represent a single SNP in estimating the effect of CD14+ monocyte on the risk of osteoporosis; the red dot represents the overall effect of CD14+ monocyte on osteoporosis evaluated by MR‐Egger method and IVW method. (E) Black dots represent the effect of a single SNP in estimating the risk of osteoporosis by CD14+/CD16− monocyte; the red dot represents the overall effect of CD14+/CD16− monocyte on osteoporosis estimated by MR‐Egger method and IVW method. (F) The black dot represents a single nucleotide polymorphism, and the SNP in the funnel diagram is basically symmetrical, so the research results are less affected by bias. (G) The abscissa indicates the influence of a single SNP on osteoporosis, and the ordinate indicates the influence of a single SNP on CD14+/CD16− monocyte. The colour line is the fitting line of the estimated causal influence of CD14+/CD16− monocyte on osteoporosis. (H) Black dots represent the effect of a single SNP in estimating the risk of osteoporosis by CD14+/CD16− monocyte; the red dot represents the overall effect of CD14+/CD16− monocyte on osteoporosis evaluated by MR‐Egger method and IVW method. (IVW, Negative variance weighting; MR, Mendel randomization; SNP, Single nucleotide polymorphism).

The causal relationship between CD14+ monocytes and CD14+/CD16− monocytes with OP was analysed using Mendelian randomization (MR). As illustrated in Table 1, the results from the inverse variance weighted (IVW) method indicate that CD14+ monocytes are positively correlated with the risk of OP. A total of 21 single nucleotide polymorphisms (SNPs) were extracted (Table S1), and the findings from the MR analysis revealed that the IVW method yielded an odds ratio (OR) of 1.1014 (95% CI: 1.0625–1.1418, *p* = 1.46E−07), the MR‐Egger method produced an OR of 1.0972 (95% CI: 1.0218–1.1781, *p* = 0.0194), and the weighted median method showed an OR of 1.1078 (95% CI: 1.0563–1.1618, *p* = 2.48E−05). These results suggest a causal relationship between CD14+ monocytes and OP. The findings are further supported by the forest and scatter plots presented in Figure 2A–D. Similarly, as shown in Table 2, CD14+/CD16− monocytes also exhibit a positive correlation with the risk of OP. A total of 22 SNPs were extracted (Table S2), and the MR analysis results indicated that the IVW method yielded an OR of 1.1029 (95% CI: 1.0651–1.1420, *p* = 3.67E−08), the MR‐Egger method produced an OR of 1.0935 (95% CI: 1.0231–1.1687, *p* = 0.0159), and the weighted median method showed an OR of 1.1044 (95% CI: 1.0570–1.1538, *p* = 8.89E−06). This also indicates a causal relationship between CD14+/CD16− monocytes and OP, with additional support from the forest and scatter plots shown in Figure 2E–H.

**TABLE 1:** Each method of Mendelian randomization assessed the causal effect of CD14+ monocyte on OP.

Method	nsnp	β	SE	*p*	lo_ci	up_ci	or	or_lci95	or_uci95
MR Egger	21	0.092743	0.036301	0.01936	0.021593	0.163893	1.097179	1.021827	1.178088
Weighted median	21	0.102396	0.024284	2.48E−05	0.0548	0.149993	1.107822	1.056329	1.161826
Inverse variance weighted	21	0.096599	0.018374	1.46E−07	0.060587	0.132612	1.101419	1.06246	1.141806
Simple mode	21	0.11255	0.044003	0.018761	0.026304	0.198797	1.119129	1.026653	1.219934
Weighted mode	21	0.105354	0.021364	8.05E−05	0.063482	0.147227	1.111104	1.06554	1.158617

**TABLE 2:** Each method of Mendelian randomization assessed the causal effect of CD14+/CD16− monocyte on OP.

Method	nsnp	β	SE	*p*	lo_ci	up_ci	or	or_lci95	or_uci95
MR Egger	22	0.089388	0.033932	0.015898	0.022881	0.155894	1.093504	1.023145	1.168702
Weighted median	22	0.099286	0.02235	8.89E−06	0.055481	0.143091	1.104382	1.057049	1.153835
Inverse variance weighted	22	0.097927	0.017786	3.67E−08	0.063066	0.132788	1.102883	1.065098	1.142008
Simple mode	22	0.117548	0.046761	0.020167	0.025896	0.209199	1.124735	1.026235	1.232691
Weighted mode	22	0.101417	0.020499	6.79E−05	0.061238	0.141596	1.106738	1.063152	1.152111

### 3.3 The Ratio of CD14+ Monocyte and CD14+/CD16− Monocyte in Peripheral Blood of OP Patients Increased

**FIGURE 3:**
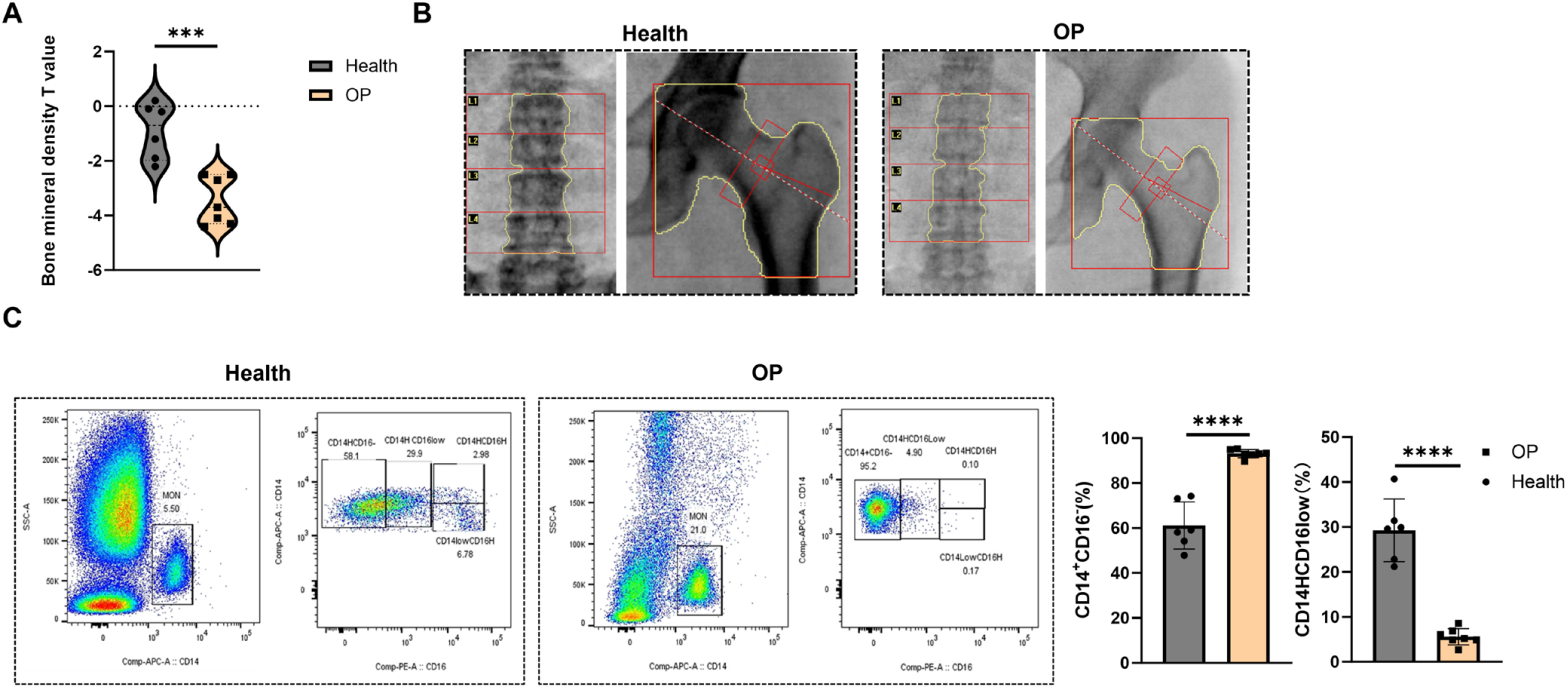
The ratio of CD14+ monocyte and CD14+/CD16− monocyte in peripheral blood of OP patients increased. (A) Comparison of bone mineral density *T*‐value in patients with healthy combination OP group. (B) Comparison of dual‐energy X‐ray in patients with healthy combination OP group. (C) Flow detection results of peripheral blood in two groups of patients. ***p < 0.001; ****p < 0.0001.

A total of thirteen clinical cases were collected, comprising six healthy individuals (three males and three females aged 30–78 years) and seven individuals in the OP group (three males and four females aged 55–91 years). There was no significant difference in baseline data between the two groups (*p* > 0.05). Compared to the healthy group, the bone mineral density *T*‐score of patients in the OP group was significantly lower (Figure 3A). Additionally, dual‐energy X‐ray absorptiometry results for the lumbar spine and hip joint indicated that bone mineral density in the OP group was markedly reduced compared to the healthy group (Figure 3B). The results of peripheral blood flow tests indicate that the ratio of CD14+ monocytes and CD14+/CD16− monocytes in the OP group is significantly higher than that in the healthy group; particularly, the difference in CD14+/CD16− monocytes is even more pronounced (Figure 3C).

### 3.4 CD14 and OP Were Positively Correlated

**FIGURE 4:**
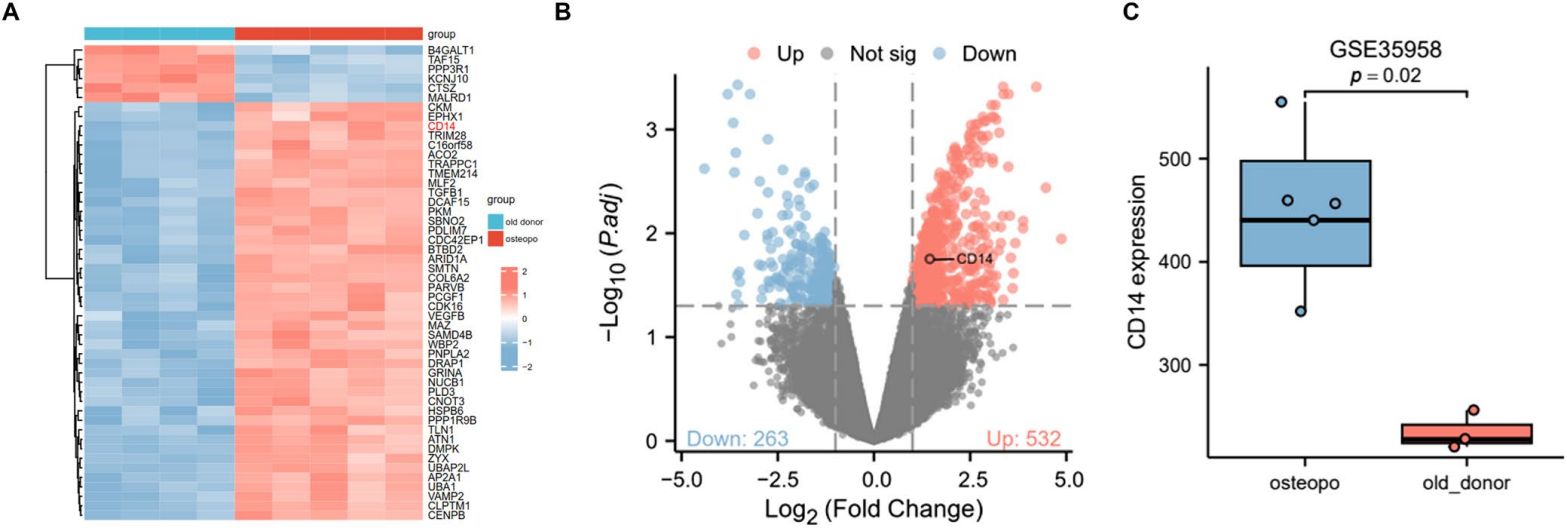
CD14 and OP are positively correlated. (A) Heatmap of 50 most significantly differentially expressed genes related to OP. (B) Volcano map of op‐related gene difference analysis. (C) Box diagram of the expression difference of CD14 gene in hMSC samples of OP group and control group.

The ratio of CD14+/CD16− monocytes significantly increased in the peripheral blood of patients with OP. We hypothesise that CD14 plays a crucial role in this process. We searched the Gene Expression Omnibus (GEO) database for gene expression datasets related to OP. The research data is sourced from the GSE35958 project in the NCBI GEO database, utilising the Affymetrix Human Genome U133 Plus 2.0 Array platform (GPL570). This dataset comprises expression profiles from human mesenchymal stem cells (hMSC), divided into two groups: the control group (hMSC‐old_donor), which includes 4 samples derived from healthy elderly donors aged 79–89 years. The treatment group (hMSC‐osteopo_donor) consists of five samples, with donors who are also elderly but suffering from osteoporosis. Linear modelling and Bayesian testing were performed using the limma package in Bioconductor. By constructing the design matrix and contrast matrix, we compared gene expression levels between two sample groups to identify differentially expressed genes (DEGs). The criteria for DEG selection were: FDR (adjusted *p*‐value) < 0.05; |log_2_FoldChange| > 1. We selected the top 50 most significantly differentially expressed genes and constructed a heatmap to illustrate their expression patterns across various samples, accompanied by annotations based on sample grouping. Our analysis revealed a significant increase of CD14 in the OP group (Figure 4A). Through differential analysis using volcano plot, it can be observed that CD14 is significantly elevated in OP (Figure 4B). The box plot we generated for further analysis indicates that CD14 is expressed significantly higher in the ‘hMSC‐osteopo_donor’ group compared to the ‘hMSC‐old_donor’ group, with a statistically significant difference (*p* < 0.05) (Figure 4C).

### 3.5 Dual Regulation of CD14 in Bone Remodelling: Affecting the Differentiation of Monocytes Into Osteoclasts and the Proliferation and Mineralisation of Osteoblasts

**FIGURE 5:**
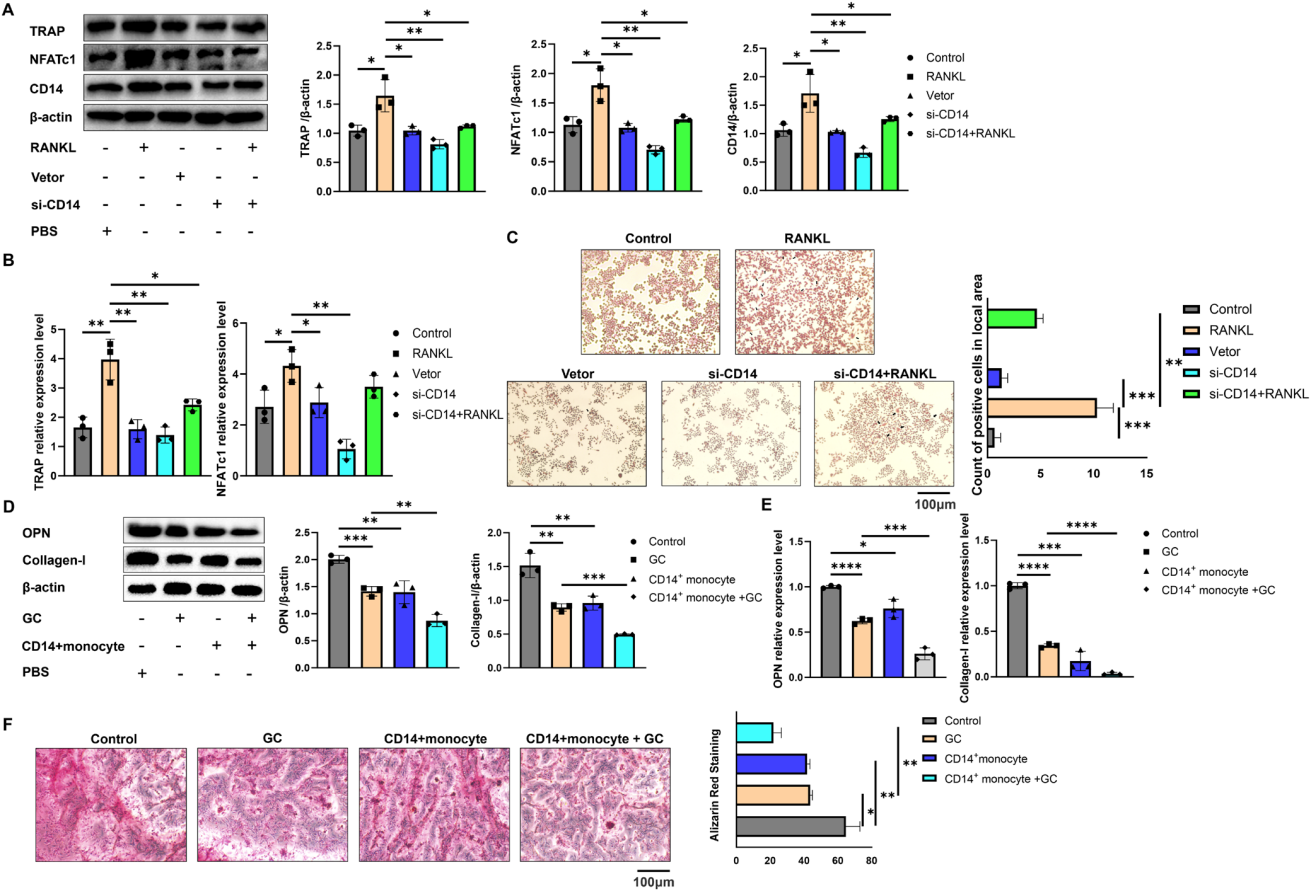
Dual regulation of CD14 in bone remodelling: Affecting the differentiation of monocytes into osteoclasts and the proliferation and mineralisation of osteoblasts. (A) The expressions of TRAP, NFATc1 and CD14 in monocytes of each group were analysed by western blot. (B) qPCR was used to analyse the mRNA expression of TRAP and NFATc1 in monocytes of each group. (C) TRAP staining was used to verify the differentiation of monocytes into osteoclasts in each group, and the black arrows represent osteoclasts. The scale is 100 μm. (D) The expression of OPN and Collagen‐I in bone cells was analysed by western blot. (E) The mRNA expression of OPN and Collagen‐I in each component bone cell was analysed by qPCR. (F) Alizarin red staining was used to analyse the mineralisation of bone cells. The scale is 100 μm. *p < 0.05; **p < 0.01; ***p < 0.001; ****p < 0.0001.

We utilised RANKL factor to stimulate monocytes and simulate an osteoporosis environment in vitro. Our findings indicate that RANKL enhances the expression levels of TRAP and NFATc1 proteins in monocytes, suggesting their transformation into osteoclasts. Notably, the expression level of CD14 protein was positively correlated with the extent of osteoclast transformation. Inhibition of CD14 expression also impeded this transformation process (Figure 5A). At the nucleic acid level, following the inhibition of CD14 expression, the mRNA levels of TRAP and NFATc1 in monocytes were similarly reduced (Figure 5B). In the TRAP staining experiment conducted on monocytes across all groups, we observed that monocytes transformed into multinucleated osteoclasts in response to RANKL stimulation; however, the inhibition of CD14 expression also effectively curtailed the transformation of monocytes into multinucleated cells (Figure 5C).

During the progression of osteoporosis (OP), the activity of osteoblasts is inhibited. Previous studies have utilised glucocorticoids (GC) to stimulate osteoblasts in order to simulate the OP model in vitro. Osteopontin (OPN) and collagen type I (Collagen‐I) are the primary markers of osteoblasts. Our findings indicate that GC intervention can significantly reduce the protein expression levels of OPN and Collagen‐I in osteoblasts. Furthermore, the co‐culture of CD14+ monocytes with osteoblasts exacerbates the biological activity of GC (Figure 5D). Similarly, GC treatment inhibits the mRNA levels of OPN and Collagen‐I in osteoblasts, and the co‐culture of CD14+ monocytes with osteoblasts further accelerates this inhibitory process (Figure 5E). Alizarin red staining, a classic assay for assessing the mineralisation capacity of osteoblasts, revealed that GC treatment diminishes the mineralisation degree of osteoblasts. Additionally, the co‐culture of CD14+ monocytes with osteoblasts further reduces the degree of mineralisation (Figure 5F).

### 3.6 CD14 Coordinates the Functions of Bone Immune Cells Through the NF‐κB and TGF‐β/SMAD3 Axis

**FIGURE 6:**
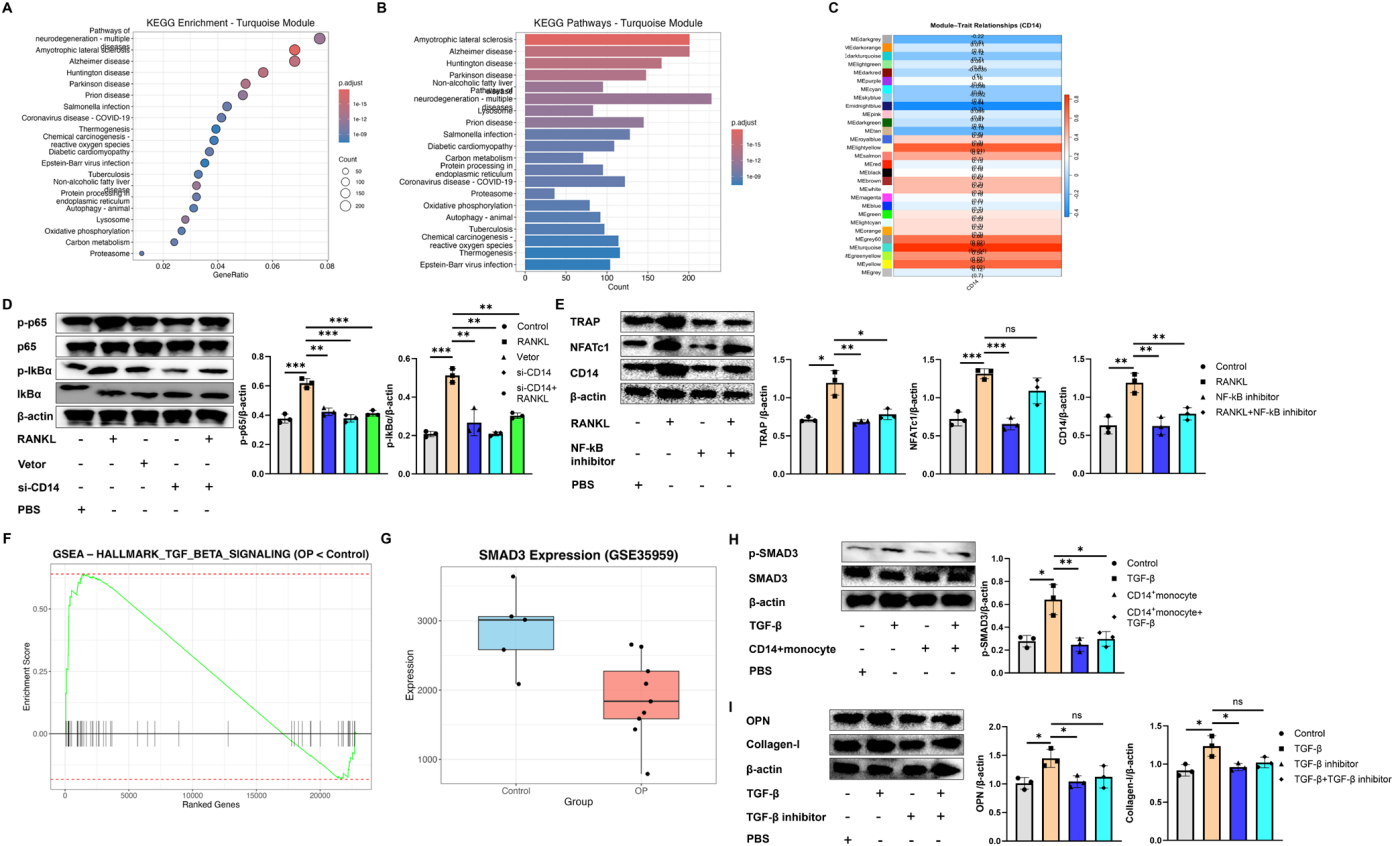
CD14 coordinates the functions of bone immune cells through the NF‐κB and TGF‐β/SMAD3 axis. (A) KEGG enrichment analysis (dotplot). (B) KEGG enrichment analysis (barplot). (C) WGCNA module‐trait correlation graph. (D) The expression of p‐p65, p65, p‐lkBα and lkBα in bone cells was analysed by western blot. (E) The expression of TRAP, NFATc1 and CD14 in bone cells was analysed by western blot. (F) Expression pattern of GSEA enrichment curve‐TGF‐β signal pathway in GSE35959. (G) SMAD3 expression box diagram‐GSE35959 data set. (H) The expression of p‐SMAD3 and SMAD3 in osteoblast was analysed by western blot. (I) The expression of OPN and Collagen‐I in osteoblast was analysed by western blot. *p < 0.05; **p < 0.01; ***p < 0.001.

This study systematically evaluates the biological functions of key modules (turquoise) through KEGG enrichment analysis (dotplot and barplot), clarifying their enrichment characteristics in immune activation, lysosomal function, ROS metabolism and osteoclast‐related pathways (Figure 6A,B). A gene co‐expression network was constructed using WGCNA, and module‐trait association analysis revealed the driving relationship between CD14 expression and immune metabolism as well as lysosomal activation programs (Figure 6C). In the NF‐κB pathway, after 5 min of RANKL stimulation, the phosphorylation levels of p65 and IκBα showed significant differences compared to the Control group. When we inhibited the expression of CD14, this phosphorylation process was similarly suppressed (Figure 6D). When we inhibit the NF‐κB pathway, the process by which RANKL promotes the protein expression of TRAP and NFATc1 in monocytes is also suppressed, and the expression level of CD14 similarly decreases (Figure 6E). Further based on the GSE35959 dataset, the GSEA method was employed to analyse the expression patterns of the TGF‐β signalling pathway. A box plot was used to illustrate the expression differences of the key downstream gene SMAD3 between the OP group and the control group (Figure 6F,G). TGF‐β intervention can activate the SMAD3 signalling pathway in osteoblasts, and co‐culturing CD14+ monocytes with osteoblasts can inhibit the phosphorylation of SMAD3 (Figure 6H). At the same time, when we inhibit the TGF‐β pathway, the protein expression levels of OPN and Collagen‐I promoted by TGF‐β in osteoblasts are significantly suppressed (Figure 6I).

## 4 Discussion

### 4.1 Osteoporosis (OP)

Osteoporosis (OP) is a systemic bone disease characterised by a decrease in bone mass, destruction of bone microstructure, and an increased risk of fractures. The global population is entering an ageing phase, with over 200 million individuals currently affected by reduced bone mineral density and OP. The prevalence of OP and associated fractures presents significant challenges to medical and health systems [26]. Osteoporosis can generally be classified into two categories: primary and secondary osteoporosis. Primary OP is the most prevalent form, which is further subdivided into type I postmenopausal osteoporosis and type II senile osteoporosis. Under physiological conditions, the dynamic equilibrium between osteoblast‐mediated bone formation and osteoclast‐mediated bone resorption is referred to as bone remodelling. Osteoporosis occurs when this balance is disrupted, leading to an increase in bone resorption and subsequent bone loss [27, 28]. Past research has established a new interdisciplinary field ‘bone immunology’ to explore the complex relationship between bones and the immune system [29].

Monocytes can be divided into three types: CD14+/CD16−, CD14+/CD16+ and CD14−/CD16+ [30, 31]. Osteoclasts, originating from the mononuclear phagocyte system, are specialised terminally differentiated cells that can fuse with their mononuclear precursor cells through various mechanisms to form large multinucleated cells [32]. There is a positive correlation between monocytes and OP. However, previous research on the relationship between monocytes and various subtypes of OP has been insufficiently comprehensive. In this study, Mendelian randomization analysis revealed a positive correlation between CD14+ monocytes and CD14+/CD16− monocytes with OP. Furthermore, clinical case analyses indicate that the ratio of CD14+/CD16− monocytes in the peripheral blood of OP patients is significantly higher than that in healthy individuals, corroborating our earlier Mendelian randomization findings. Additionally, we hypothesise that CD14 may be a key factor influencing the transformation of monocytes into osteoclasts. A search of the GEO database for OP‐related data revealed an increased expression of CD14 in the peripheral blood of OP patients. Moreover, our osteoclast cell biology experiments indicate that the inhibition of CD14 expression in monocytes can prevent their differentiation into osteoclasts by suppressing the NF‐kB pathway. However, the disruption of the dynamic balance between osteoclasts and osteoblasts is primarily responsible for OP. Thus, we further investigated whether CD14+ monocytes affect the proliferation and mineralisation of osteoblasts. Our findings revealed that co‐culturing CD14+ monocytes with osteoblasts obstructed the TGF‐β/SMAD3 signalling pathway in osteoblasts, thereby inhibiting their proliferation and mineralisation processes. This supports the causal relationship between CD14+ monocytes or CD14+/CD16− monocytes and osteoclastogenesis, through their interactions with osteoclasts and osteoblasts.

This study aligns with previous research; however, it diverges by revealing that not all monocytes exhibit a clear causal relationship with OP through Mendelian randomization analysis. Specifically, only CD14+ monocytes and CD14+/CD16− monocytes demonstrate a significant causal relationship with OP. Furthermore, our clinical analysis revealed that CD14+/CD16− monocytes play a more significant role in OP, leading us to speculate that CD14 may be an important target gene for OP treatment. We further validated the reliability of our results through in vitro simulation experiments involving osteoclasts and osteoblasts within the context of OP.

However, this study has several limitations: (1) The research data source exhibits regional limitations, as the majority of individuals in the GWAS summary data are of European descent, potentially impacting the generalizability of the results. (2) The OP patients were not stratified by age, sex, or various health statuses, which may render the findings less specific. Future enhancements to the GWAS database could yield more precise results. (3) The range of CD14+ monocytes is relatively broad. In our flow cytometry analysis, we categorised monocytes into four types: CD14HCD16−, CD14HCD16low, CD14HCD16H and CD14lowCD16H. We observed a significant increase in the proportion of the first monocyte type in the peripheral blood of OP patients. Based on Mendelian randomization and flow cytometry results from clinical patient samples, we identified CD14 as a crucial influencing factor, which may account for the observed heterogeneity in Mendelian randomization outcomes.

This study provides significant insights into the causal relationship between exposure and outcome by utilising a large sample of GWAS data. This approach minimises the influence of confounding factors and reverse causal relationships, thereby offering valuable information for disease prevention and intervention measures at the genetic level. Furthermore, the reliability of the results is validated through clinical and cytological experiments, which is essential for understanding the pathological interactions between immune cells and bone health. Building upon previous research, we have confirmed that two subtypes of monocytes (CD14+ monocytes or CD14+/CD16− monocytes) exhibit a causal relationship with OP. Additionally, CD14 is identified as a crucial factor influencing the differentiation of monocytes into osteoclasts, and CD14+ monocytes may even impact the mineralisation process of osteoblasts. These findings provide a reference point for future prevention and treatment strategies for OP. It is possible that blood tests could be utilised to quantify the corresponding OP risk, and targeted therapy aimed at CD14 may emerge as a novel approach to OP treatment.

## 5 Conclusion

There exists a clear causal relationship between CD14+ monocytes or CD14+/CD16− monocytes and OP. This relationship is contingent upon the role of CD14 in regulating the transformation of monocytes into osteoclasts and promoting the proliferation and mineralisation of osteoblasts.

## Author Contributions

Haoran Wang and Ping Zhou wrote the manuscript. Xiao Ma and Jie Zhang designed the study and collected and analysed the data. Boyao Wang and Jun Liu gave final approval of the version to be published. All authors have agreed on the journal to which the article has been submitted. Haoran Wang, Ping Zhou and Xiao Ma contribute equally to this article.

## Funding

This study was supported by the Jiangsu Provincial Commission of Health and Family Planning, “Six One” Project of Jiangsu province LGY2016018.

## Ethics Statement

All clinical samples and case data were obtained from the Department of Orthopaedics at the Second Affiliated Hospital of Nanjing Medical University. This study received approval from the Ethics Committee of the Second Affiliated Hospital of Nanjing Medical University, with the ethical document number 2025‐KY‐002‐01.

## Consent

All authors agree to the submission and publication of the manuscript.

## Conflicts of Interest

The authors declare no conflicts of interest.

## Supporting information


**Table S1:** jcmm71024‐sup‐0001‐TableS1.xlsx.

**Table S2:** jcmm71024‐sup‐0002‐TableS2.xlsx.

## Data Availability

The authors have nothing to report.
